# Treatment of Advanced Non-small-Cell Lung Cancer with Qi-Nourishing Essence-Replenishing Chinese Herbal Medicine Combined with Chemotherapy

**DOI:** 10.1186/s12575-018-0074-9

**Published:** 2018-04-01

**Authors:** Yabin Gong, Zhenye Xu, Changjuan Jin, Haibin Deng, Zhongqi Wang, Weidong Zhou, Ming Zhang, Xiaozhen Zhao, Lifang Wang

**Affiliations:** 1 Department of Oncology, Longhua Hospital, Shanghai University of Traditional Chinese Medicine, Shanghai, ,200032 China; 2 Department of Integrated Chinese and Western Medicine, Shanghai Chest Hospital, Shanghai, ,200030 China; 3 Department of Oncology, Yueyang Hospital of Integrated Traditional Chinese and Western Medicine, Shanghai University of Traditional Chinese Medicine, Shanghai, ,200437 China; 4 Department of Oncology Longhua Hospital, Shanghai University of Traditional Chinese Medicine, 725 Southern of Wan Ping Road, Shanghai, China

**Keywords:** Qi-nourishing essence-replenishing, Chinese herbal medicine, Essence and qi deficiency, Chemotherapy, Non-small-cell lung cancer

## Abstract

**Background:**

To evaluate the effect of qi-nourishing essence-replenishing Chinese herbal medicine combined with chemotherapy in survival of advanced non-small-cell lung cancer(NSCLC) patients with essence and qi deficiency.

**Methods:**

A prospective multi-centered randomized controlled study was conducted, and 266 advanced NSCLC patients were enrolled. 126 patients in control group received Vinorelbine plus cisplatin(NP) chemotherapy combined with symptom-oriented Chinese herbs medication(without qi-nourishing essence-replenishing herbs);140 patients in experimental group received NP chemotherapy combined with qi-nourishing essence-replenishing Chinese herbal medication(Kangliu Zengxiao Decoction and modified Feiyanning Decoction, during and after chemotherapy respectively).

**Results:**

One patient in control and 2 in experimental group were excluded for failure to complete two cycles of chemotherapy. During follow-up, 17 and 7 patients in control and experimental group were excluded respectively(4 and 4 for taking Gefetinib after disease progression, 4 and 2 for receiving other chemotherapeutic regimens, 9 and 1 for lost to follow-up). 239 patients were included in the final analysis (131 in experimental group and 108 in control). Median overall survival in experimental group was significantly longer than control group (14.87vs.12.97 months,*P* = 0.027). In experimental and control group, 1-year, 3-year, 5-year, 7-year, and 9-year survival rates were 57% vs. 53%, 17% vs. 8%, 10% vs. 2%, 6% vs. 0%, and 6% vs. 0%, respectively.

**Conclusion:**

Qi-nourishing essence-replenishing Chinese herbal medicine combined with chemotherapy improves survival of advanced NSCLC patients with essence and qi deficiency.

## Background

Lung cancer is one of the malignancies with highest morbidity and mortality in China [1]. Approximately 75–85% of lung cancers are non-small cell lung cancers (NSCLC), including squamous carcinoma, adenocarcinoma and large cell carcinoma [2]. Most patients (70%) are diagnosed with advanced disease [3]. Although with the advent of the third-generation chemotherapeutic drugs (gemcitabine, docetaxel, and pemetrexed), the clinical efficacy remains limited. Indeed, the overall survival of advanced NSCLC patients remains low after first-line chemotherapy. Prognosis for these patients is poor with 5-year survival rates reported as less than 5% [4]. Platinum-based doublet chemotherapy is the standard first-line treatment for advanced NSCLC when genomic testing reveals no activating epidermal growth factor receptor (EGFR) mutations, anaplastic lymphoma kinase (ALK) or ROS1 rearrangement [5], with objective response rates between 15~ 30% [6, 7]. Since chemotherapy have been adopted in China, Chinese herbal medicine(CHM) is widely used in combination with chemotherapy to cope with chemotherapy-related side effects as well as prolonging the survival time of patients. Our previous studies have demonstrated that qi-nourishing essence-replenishing CHM combined with chemotherapy can improve short-term lesion stability, quality of life and immunity in advanced NSCLC patients [8–12]. However, its effect on the long-term survival remains unknown.

According to TCM theory, qi and essence are the fundamental substance constituting human body and maintaining physical activities [13–15]. The deficiency of qi and essence leads to malfunction of the body, with clinical manifestation of fatigue and lassitude, aching lumbus and limp legs, dizziness and tinnitus, sensitivity and intolerance to cold, spontaneous perspiration and night sweats, thirst or reduced water intake, frequent night-time urination, thready or weak pulse, red or pink tongue with some tongue coating. The pathology of lung cancer, according to TCM, is the deficiency of qi and essence and the pathogenic factors resulting from it. Therefore, the key point of TCM cancer treatment is to strengthen qi and essence and eliminate pathogenic factors, and qi-nourishing essence-replenishing CHM has been proved with satisfactory short-term effect [9].

In this study, a perspective randomized single-blinded clinical trial was conducted to evaluate the survival benefit of qi-nourishing essence-replenishing CHM combined with Vinorelbine + Cisplatin (NP) regimen in advanced NSCLC patients with essence and qi deficiency, in comparison to symptom-oriented CHM (without qi-nourishing essence-replenishing herbs) with NP regimen. The findings should help to prolong the overall survival time for advanced NSCLC patients.

## Methods

### Patient Selection

#### Source of Patients

Patients admitted in 1) Department of Oncology, Longhua Hospital, Shanghai University of Traditional Chinese Medicine; 2) Department of Integrated Traditional Chinese and Western Medicine,Shanghai Pulmonary Cancer Clinical Medicine Center, Shanghai Chest Hospital; 3) Department of Oncology, Shanghai Pulmonary Hospital, who received treatments between January 2006 and December 2012, were enrolled. The protocol was approved by Shanghai Pudong food and drug administration,All participants signed informed consent forms.

#### Inclusion Criteria

1. definitive diagnosis of NSCLC based on imaging, pathological, or cytological evidence;
2. diagnosis of inoperable stage IIIb-IV or postoperative recurrent metastatic NSCLC based on the Union for International Cancer Control (UICC) TNM Classification for Malignant Tumors (6th edition) [16];
3. Eastern Cooperative Oncology Group (ECOG) performance status (PS) score ≤ 2 with expectation to complete 2–4 cycles of chemotherapy and survive more than 4 months;
4. aged between 18 and 75 years;
5. matching the traditional Chinese medicine (TCM) syndrome differentiation of essence and qi deficiency based on the TCM syndrome differentiation of primary bronchial lung cancer in the Guidelines of clinical research on Chinese new herbal medicine [17]:

The main symptoms/signs included: a) fatigue and lassitude; b) aching lumbus and limp legs; c) dizziness and tinnitus; d) sensitivity and intolerance to cold; e) red or pink tongue with some tongue coating.

Secondary symptoms/signs included: a) spontaneous perspiration and night sweats; b) thirst or reduced water intake; c) frequent night-time urination; d) thready or weak pulse.

A definitive diagnosis was made for any patient showing at least three main and one secondary symptoms/signs, or two main and two secondary symptoms/signs.

#### Exclusion Criteria

1. unfulfillment of inclusion criteria;
2. severe complications of heart, liver, kidney and/or blood diseases;
3. severe infections;
4. failure to complete the 2 chemotherapeutic courses;
5. PS score>2;
6. not matching the TCM syndrome differentiation of essence and qi deficiency.

#### Case Exclusion Criteria

1. violating the protocol criteria;
2. not matching the inclusion criteria but enrolled.

#### Withdrawal Criteria

1. receiving any targeted therapy or any other chemotherapeutic regimen due to disease progression within 3 months after study initiation;
2. exhibiting poor compliance that affected efficacy and safety assessments during the study process;
3. unsuitable to continue the study due to severe adverse events, complications or special physiological changes;
4. less than 3 months of Feiyanning Decoction administration after chemotherapy, or withdrawing from the study at patient’s own discretion;
5. administering combined medications that potentially affect efficacy and safety assessments of the study medication;
6. withdrawing, lost to follow-up, or dying due to disease-irrelevant reasons before study completion.

#### Criteria for Study Termination

This study would be terminated in any of the following situations:

1. patients suffered from any severe adverse events;
2. any major deficiencies in the clinical protocol or significant deviation was determined to pose difficulties for evaluation.

### Study Methods

#### Randomization

Patients were randomized into the experimental and control groups using complete randomization with SPSS16.0 software.

#### Blinding

Subjects were single-blinded to grouping.

#### Source and Preparation of Medications

1) Kangliu Zengxiao Decoction(Table 1) and Feiyanning Decoction(FYN decoction Table 2).

**Table 1:** Standard formula of Kang Liu Zeng Xiao decoction

Chinese name	Pharmaceutical name	Crude drug content (g)
Huang Qi	*Astragalus mongholicus*	30
Huang Jing	Polygonatum sibiricum	30
Jiao Gu Lan	Gynostemma pentaphyllum	15
Ling Zhi	Ganoderma lucidum	24
Cang Zhu	Atractylodes chinensis	9
Nv Zhen Zi	*Ligustrum lucidum*	15
Huang Lian	Coptis chinensis	6

**Table 2:** Standard formula of Fei Yan Ning decoction

Chinese name	Pharmaceutical name	Crude drug content (g)
Sheng Huang Qi	Astragalus mongholicus	40
Huang Jing	Polygonatum sibiricum	30
Shan Zhu Yu	Cornus Oycinalis	15
Ling Zhi	Ganoderma lucidum	30
Yin Yang Huo	Epimedium davidii Franch	30
Nv Zhen Zi	Ligustrum lucidum	15
Bai Zhu	Rhizoma Atractylodis Macrocephalae	9
Feng Fang	Comb	9
Shan Ci Gu	Pseudobulbus Cremastraeseu Pleiones	15
Gan Chan Pi	Toad Skin	9
Chong Lou	Paris polyphylla Smith	15
Shi Jian Chuan	Chinese Sage Herb	30

Kangliu Zengxiao Decoction(KLZX decoction) is composed of *Astragalus mongholicus*, Ganoderma lucidum, Coptis chinensis, Atractylodes chinensis, etc. Feiyanning Decoction(FYN decoction) contains Astragali Radix, *Ganoderma lucidum*, *Polygonatum sibiricum*, Herba Epimedii, Paris polyphylla Smith, beehive, and dried toad skin. All herbs were provided by Longhua Hospital pharmacy, dissolved in 1000 ml water, soaked for 30 min, decocted to 200 ml with gentle heat; then these herbs were dissolved in 500 ml water and decocted to 100 ml. Two decoctions(300 ml) were mixed, and aliquoted into 2 plastic bags (150 ml each), ready for use. The crude drug content was 1.0 g/ml.

2) Chemotherapeutic agents.

Vinorelbine (NVB) was provided as 10 mg/bottle (Pierre Fabre, France). Cisplatin (DDP) was obtained as 20 mg/bottle (Qilu Pharmaceutical, China).

#### Methods of Administration

##### 1) Control Group

The NP regimen, comprising NVB (25~ 30 mg/m^2^, day 1, day 8) and DDP (70~ 80 mg/m^2^, divided into day 1–3) was used once every 3 weeks for 2–4 cycles. 150 ml of modified Jupi Zhuru Decoction (Table 3)were used during the first treatment period(chemotherapeutic period), while 150 ml of symptom-oriented decoction (Table 4) was administered during the second treatment period(14 days after the last course of chemotherapy). The symptom-oriented decoction was completely different from that in the expeimental group, without qi-nourishing essence-replenishing herbs. The basic principles of the modification included: in patients with diarrhea, lentils and dioscorea were added; in thirsty ones, *Glehnia littoralis*, Cochinchinese Asparagus and *Ophiopogon japonicus*; for those with cough and bitter almond, *Eriobotrya japonica* and Rhizoma phragmitis were added; for those having trouble sleeping, Caulis Polygoni Multiflori and *Albizia julibrissin* were added; in subjects with lower back soreness, Dipsacus root, Herba Taxilli and *Eucommia ulmoides* were added; in those with palpitation, honey-fried licorice root and Salvia miltiorrhizae were added; in patients with poor appetite, chicken gizzard membrane, rice sprout and malt etc. were added.

**Table 3:** Standard formula of modified Jupi Zhuru decoction

Chinese name	Pharmaceutical name	Crude drug content (g)
Dang Shen	*Codonopsis pilosula*	15
Chen Pi	Pericarpium citri reticulatae	9
Jiang Ban Xia	*Pinellia ternata*	9
Jiang Zhuru	*Bambusa tuldoides* Munro	9
Da Zao	Zizyphus jujuba	9
Gan Cao	Glycyrrhiza uralensis	6

**Table 4:** Standard formula of symptom-oriented decoction

Chinese name	Pharmaceutical name	Crude drug content (g)
Xing Ren	Apricot kernel	9
Lu Gen	Reed rhizome	30
Pi Pa Ye	Folia eriobotryae	9
Dan Shen	Salvia miltrorrhiza	15
Bai Hua She She Cao	Herba hedyotis	30
Pu Gong Ying	Dandelion	30
Yu Xing Cao	*Houttuynia cordata*	30
Jin Yin Hua	Honeysuckle	9
Chen Pi	Pericarpium citri reticulatae	9
Xia Ku Cao	Prunella spike	15
Sheng Mu Li	Concha ostreae	30

##### 2) Experiment Group

The regimen, dose, usage, and course of the chemotherapy were the same as control group. 150 ml KLZX decoction was orally administered twice daily during the first treatment period (chemotherapeutic period), and within14 days after the last course of chemotherapy. During the second treatment period(14 days after the last course of chemotherapy), 150 ml of modified FYN decoction was administered twice daily for one year or until intolerable side effect.

The basic principles of the modification as control group.

#### Outcome Measure

The main outcome measure in this study was overall survival.

### Statistical Analysis

The SPSS16.0 software was used for statistical analysis. Quantitative data were expressed as mean ± SD, and compared by *t* test; qualitative data were expressed as rate, and analyzed by the χ^2^ or Mann-Whitney test. Kaplan-Meier curves were generated to assess survival, with the Log-rank test adopted for comparisons.

## Results

### General Information

The 266 subjects matching the inclusion criteria were enrolled and treated between January 2006 and December 2012. The last follow-up was December 2015, and overall follow-up rate was 90.2%. One and two patients respectively in the control and experimental groups were excluded for failing to complete the two chemotherapeutic courses. Another 17 patients were further excluded in control group during follow-up, including 4, 4, and 9 patients for taking Gefetinib due to disease progression, administering other chemotherapeutic regimens, and lost to follow-up, respectively; in experimental group, 7 patients were further excluded, including 4, 2, and 1 patients, respectively, for taking Gefetinib due to disease progression, administered other chemotherapeutic regimens, and lost to follow-up. Therefore, 239 were included in the final analysis, with 131 and 108 in the treatment and control groups respectively. No significant difference was found in baseline characteristics (gender, age, staging and histology) between the two groups (Table 5).

**Table 5:** baseline characteristics**.** No significant difference was found in baseline characteristics (gender, age, staging and histology) between the two groups

Characteristic	Treatment group(*n* = 131)	Control group(*n* = 108)	*P* value
Sex-n(%)			0.551
Male	80(61.06)	66(61.11)	
Female	51(38.94)	42(38.89)	
Age			0.889
Median(years)	60.89(40–78)	60.56(41–80)	
< 65 (%)	89(54.3)	75(56)	
≥ 65 (%)	42(45.7)	33(44)	
Disease stages at entry-n(%)			0.367
IIIa	2(1.5)	4(3.7)	
IIIb	35(26.7)	34(31.5)	
IV	94(71.8)	70(64.8)	
Histology-n(%)			0.763
Adenocarcinoma	87(66.4)	74(68.5)	
Squamous carcinoma	29(22.1)	22(20.4)	
Adenosquamous carcinoma	1(0.8)	0(0)	
Large cell carcinoma and other	14(10.7)	12(11.1)	

### Survival Analysis

The median survival time (MST) was 14.87 (95% CI 11.729–18.011) and 12.97 (95% CI 11.252–14.688) months in the experiment and control groups, respectively. (*P* = 0.027) (Table. 6 and Fig. 1);1-, 3-, 5-, 7-, and 9-year survival rates were 57%,17%,10%,6%,6% in the experiment. However, 53%,8%,2%,0%,0% in control group.1-year, 3-year, 5-year, 7-year, and 9-year survival rates were improved in experiment group(Table. 7).

#### Median Survival

**Table 6:** Comparison of median survival between the two groups. Log-rank test was used to compare survival between the two groups. If patients receive qi-nourishing essence-replenishing TCM after chemotherapy for more than three months and switch to Gefetinib due to disease progression, survival was calculated as time to the first day of Gefetinib administration plus one month

Group	Median survival (month)	95% confident interval (month)	*P* value
Control	12.970 ± 0.877	11.252–14.688	0.027
Experiment	14.870 ± 1.602	11.729–18.011	

#### 1-, 3-, 5-, 7-, and 9-Year Survival Rates

**Table 7:** Comparison of 1-, 3-, 5-, 7-, and 9-year survival rates between the two groups. 1-year, 3-year, 5-year, 7-year, and 9-year survival rates were improved in experiment group

Group (%)	1-year (%)	3-year (%)	5-year (%)	7-year (%)	9-year (%)
Control	53	8	2	0	0
Experiment	57	17	10	6	6

#### Comparison of Survival Rates

**Fig. 1:**
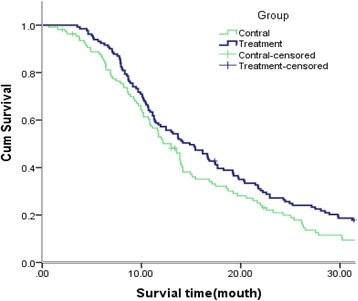
Kaplan-Meier curves for survival and cumulative survival assessment. Control and experimental groups were assessed. Control-censored data, treatment-censored data; length of survival (months)

## Discussion

Lung cancer is the malignancy with highest morbidity and mortality worldwide [18], and it is urgent to improve survival in patients with advanced lung cancer. The Vinorelbine + cisplatin (NP) regimen adopted in this study is relatively low-cost, and serves as one of the first-line standardized regimens for advanced NSCLC patients without driving gene mutations such as *EGFR* and *ALK* [19]. But its effect is far from satisfactory, and combination therapy with other agent to improve survival is in urgent need.

It is recorded in Naniing, a classical book of TCM written more than two thousand years ago, that ‘treating physical weakness by warming therapy for nourishing qi and treating essence deficiency by supplying tasty and nutritional animal and plant food’, ‘for those with lung damages nourishing their qi’, and ‘for those with renal damage nourishing their kidneys’. Professor Zhenye Xu proposed the qi-nourishing essence-replenishing antitumor therapy for advanced lung cancer. Emphasizing qi-nourishing essence-replenishing methods that tonify kidney in the treatment of advanced lung cancer is based on the following TCM theory. Kidney essence is the congenital foundation and root of functional movement of human body, and the relationship between the kidney and lung is mutual generation. Spleen spirit is the postnatal foundation, and supplementing spleen nourishes lung. Therefore, nourishing the lung, spleen and kidney, so as to nourish qi and replenish essence can correct systemic deficiency. The qi-nourishing essence-replenishing method is a therapeutic technique based on the epidemiological characteristics of lung cancer, qi-blood-fluid theory, and visceral manifestation theory, and improves clinical outcome of lung cancer.

KLZX decoction is a TCM formula developed by Prof. Zhenye Xu based on the therapeutic rules of replenishing qi and replenishing essence in combination with activating the spleen to clear heat and dissipate dampness. KLZX decoction can reduce toxicity and enhance efficacy during the chemotherapeutic period. It is mainly composed of Astragali Radix, *Polygonatum sibiricum*, *Ganoderma lucidum*, *Coptis chinensis* Franch, and *Atractylodes lancea*. Astragali Radix is sweet, mildly warm, and important herb for replenishing qi. *Polygonatum sibiricum* is a sweet and neutral herb that tonifies deficiency, neutralizes heat or cold, and replenishes essence and marrow, and therefore invigorates the kidney, replenishes essence, and nourishes the lung and spleen. *Ganoderma lucidum* nourishes and strengthens the body, benefits blood and qi, and replenishes essence and marrow. *Atractylodes lancea* is an important herb for invigorating the spleen and eliminating dampness. *Coptis chinensis* can clear heat and dampness in the middle energizer, exerting an effect of invigorating the spleen to eliminate dampness and regulating the stomach.

FYN decoction [8], a Fuzheng and pathogen-eliminating decoction used after the chemotherapeutic period, is expected to treat both the symptoms and root causes, prolonging cancer patient survival via its components of qi-nourishing essence-replenishing kidney-tonifying Chinese herbs and antitumor detoxifying pathogen-eliminating Chinese herbs.

Our previous studies [8–12] have demonstrated that therapy of qi-nourishing essence-replenishing CHM by stages combined with chemotherapy can improve short-term lesion stability, quality of life and immunity in advanced NSCLC patients.The present study demonstrated that it also prolongs overall survival and improves the 1-, 3-, 5-, 7-, and 9-year survival rates.

Our study showed that the efficacy of CHM therapy as modified KLZX decoction in the first treatment period for toxicity reducing and efficacy enhancing [11],while modified FYN decoction in the second treatment period as a maintenance regimen is non-inferior to pemetrexed, without adverse events such as nausea, vomiting, and bone marrow suppression [20, 21]. In addition, it has lower costs compared with pemetrexed. The 5-year survival rate observed in this study (10%) was significantly higher than that reported in other studies (2%), and a 9-year survival rate of 6% was obtained. However, evidences from controlled randomized clinical trials are required to compare modified FYN decoction and pemetrexed for maintenance chemotherapy.

## Conclusion

3.1 Qi-nourishing essence-replenishing CHM combined with NP chemotherapy prolongs median survival in advanced NSCLC patients with essence and qi deficiency.

3.2 Qi-nourishing essence-replenishing CHM combined with NP chemotherapy improves the 1-, 3-, 5-, 7-, and 9-year survival rates of advanced NSCLC patients with essence and qi deficiency.

## Abbreviations

ALK: Anaplastic Lymphoma Kinase; NP: Vinorelbine + Cisplatin;
CHM: Chinese Herbal Medicine; NSCLC: Non-Small-Cell Lung Cancer;
ECOG: Eastern Cooperative Oncology Group;
EGFR: Epidermal Growth Factor Receptor;
KLZX: Kangliu Zengxiao Decoction;FYN:Feiyanning Decoction;
NVB: Vinorelbine; DDP:Cisplatin.
PS: Performance Status;TCM:Traditional Chinese Medicine;
